# Identifying State-Wide Health Disparities in Diabetes Mellitus Management in Arizona: Review of Healthy People 2020 Performance

**DOI:** 10.7759/cureus.12993

**Published:** 2021-01-29

**Authors:** Katelyn Urban, Shiva Malaty, Alethea Turner, Priya Radhakrishnan

**Affiliations:** 1 Dermatology, Lake Erie College of Osteopathic Medicine, Greensburg, USA; 2 Internal Medicine, HonorHealth, Scottsdale, USA; 3 Family Medicine, HonorHealth, Scottsdale, USA

**Keywords:** public health policy, healthy people 2020, arizona, health care disparities, diabetes mellitus management

## Abstract

Healthy People (HP) 2020 is a national initiative focused on health promotion and disease prevention. Three state-wide metrics in Arizona were evaluated including diabetes-related death rate, biannual measurement of glycosylated hemoglobin (HA1c), and annual foot exam. The overall results of this review point to an alarming level of disparity that exists among Black patients for the three analyzed metrics. The American Indian population also experienced disproportionately high rates of diabetes-related death in Arizona. Additional variances were noted among uninsured patients and those who had received less than a high school degree. Identifying groups who are the most vulnerable to health inequity, investigating root cause, and addressing social determinants of health are critical to improving the health of our nation.

## Introduction and background

Diabetes mellitus (DM) affects millions of Americans, and in Arizona there are more than 600,000 people diagnosed with the disease [1]. This review identifies groups within Arizona who disproportionately received substandard medical care for their diabetes and who experienced greater diabetes-related death. Analysis of data from Healthy People (HP) 2020 highlights race, education, insurance status, and location as important factors affecting diabetes-related monitoring and outcomes. Effort to better understand why certain groups are more vulnerable than others, is necessary to reduce the significant burden that diabetes imposes on patients and the health care system. We report these findings in order to raise awareness of disparities existing in diabetes care, so that further work can be done to achieve health equity.

This review examined publicly-shared data on the Healthy People website (HealthyPeople.gov) from HP 2020. The search focused on Arizona data related to diabetes objectives for which five metrics were available. We examined the diabetes-related death rate per 100,000 (age adjusted) [2] along with metrics that were directly related to provider-monitoring of diabetes progression: increasing the proportion of adults aged 18 years and over with diabetes who have a glycosylated hemoglobin (HA1c) measurement at least twice a year and who receive at least one annual foot examination [3,4]. Data from diabetes-related deaths were categorized by sex, race, age, and location (metropolitan versus non-metropolitan). Data from the HA1c and annual foot examination objectives were divided by sex, race, age, education, household income, obesity, health insurance, and marital status. Arizona records were available from 2013, 2014, 2015, and 2017, however, data from the above subcategories were limited in certain years. Information recorded in 2017 was the most inclusive and recent, and therefore included in this review. Disparity was then calculated as the difference between recorded data from Arizona and the average from all other reporting states in 2017. HP 2020 data for diabetes-related deaths were collected from Bridged-race Population Estimates and National Vital Statistics System-Mortality. Data for the provider-monitored objectives reviewed in this article were collected via survey by the Behavioral Risk Factor Surveillance System (BRFSS). The survey is conducted annually by telephone and more than 350,000 adults are interviewed each year [5]. The pertinent questions asked of patients who identified as having been diagnosed with diabetes include, “About how many times in the past 12 months has a health professional checked your feet for any sores or irritations” and, “A test for ‘A one C’ measures the average level of blood sugar over the past three months. About how many times in the past 12 months has a doctor, nurse, or other health professional checked you for ‘A one C’?”

## Review

**Figure 1:**
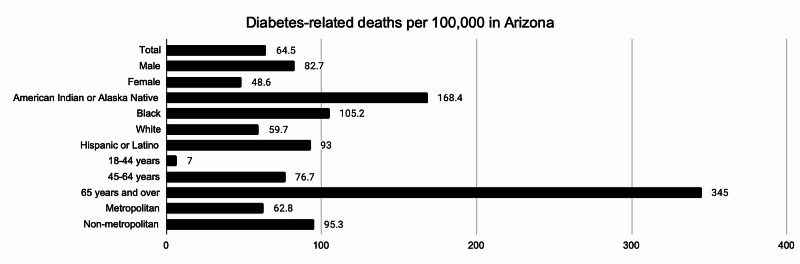
Diabetes-related deaths per 100,000 in Arizona

The diabetes-related death rate in Arizona was lower than that of the United States. However, certain populations within Arizona did not meet the national target (66.6 per 100,000) for this initiative (Figure 1). Individuals identifying as “American Indian or Alaska Native” had the highest diabetes-related death rate among races at 168.4 per 100,000, followed by those who identified as “Black or African American” and “Hispanic or Latino,” at 105.2 and 93, respectively. Individuals identifying as “White” had a death rate of 59.7. Those living in a non-metropolitan area had a higher rate than those living in a metropolitan area at 95.3 versus 62.8, respectively. Not surprisingly, the highest death rate was seen in the “65 years and over” group. Individuals identifying as “American Indian or Alaska Native” and “Black or African American” also had the highest diabetes-related death rates nationally at 94.6 and 103.6, respectively [2].

**Figure 2:**
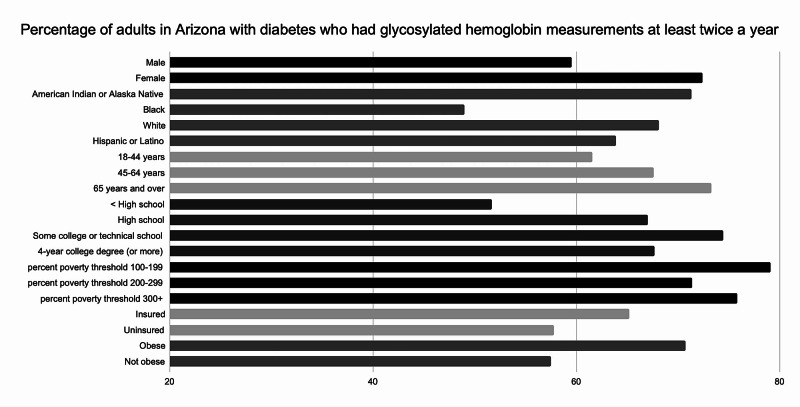
Percentage of adults in Arizona with glycosylated hemoglobin (HA1c) measurements at least twice a year

Arizona data linked to the HP 2020 objective to increase the proportion of adults with diabetes who have a HA1c measurement at least twice per year were evaluated (Figure 2). Including the District of Columbia, 39 of 51 states were represented in the data. Of all reporting states, 70.7% of adult patients with diabetes achieved the above-stated goal. In Arizona, only 65.4% met this metric. Further analysis of state-level data reveals that Black people received less testing compared to other groups in Arizona. Of patients identified as “Black or African American only,” 49% with diabetes received biannual HA1c testing compared to 71.3% of “American Indian or Alaska Native only,” 68.1% of “White only,” and 63.9% of “Hispanic or Latino” patients. Among the other analyzed categories, no other subgroup fell below 50%. Another identified group receiving inadequate HA1c testing was among patients aged 25 years and older who had attained less than a high school degree. In 2017, 51.7% of these patients in Arizona received biannual HA1c compared to 69.7% of patients who had attained high school education or beyond. Analysis of socio-economic status in Arizona revealed the group living at 200%-299% poverty threshold had the lowest attainment of this goal with 71.4% of patients having HA1c testing at least twice a year. This observation was reversed at the national level with the highest proportion of testing being done in the group living at 200%-299% poverty threshold at 79%. Analysis of racial disparities at the national level revealed individuals identifying as “Hispanic or Latino” with the least amount of testing at 60.2%. Having less than a high school degree was also correlated to worse outcomes nationally, as only 58.8% of individuals reporting less than a high school degree reached this target [3]. 

**Figure 3:**
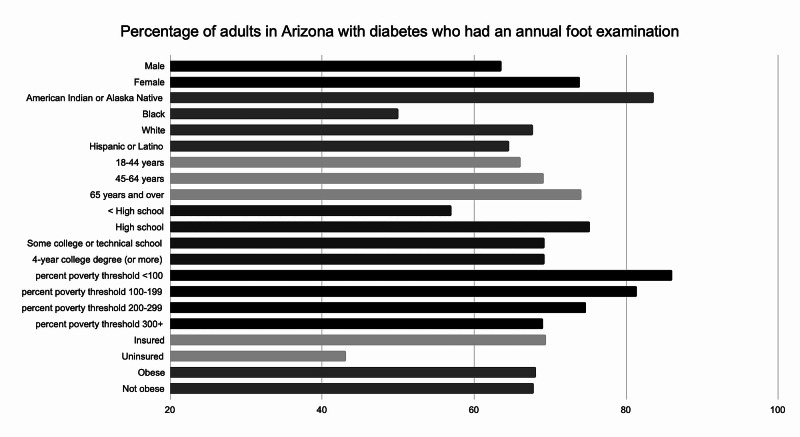
Percentage of adults in Arizona with diabetes who had an annual foot examination

Among all reporting states, the average percentage of adults with diabetes who had an annual foot examination in 2017 was 69.8%. As with the metric previously discussed, 39 of 51 states, including the District of Columbia, were represented in the data. In contrast, only 68.4% of Arizona patients were reported to have met this metric (Figure 3). Further analysis of state-level data reveals that Black patients, as well as patients without health insurance, had fewer documented foot exams compared to other subgroups. 50% of patients identified as “Black or African American only,” had an annual foot exam compared to 83.6% of “American Indian or Alaska Native only,” 67.7% of “White only,” and 64.6% of “Hispanic or Latino” patients. Furthermore, evaluation of health insurance status reveals that 43.1% of uninsured patients had an annual foot exam compared to 69.5% of insured patients. Socioeconomic status also impacted outcomes, evidenced by the decline in the percentage of people reaching this set target as the percent poverty threshold increased. A similar trend was observed nationally with 64.8% of patients living at a 200%-299% poverty threshold receiving an annual foot exam. Similar to Arizona, a large disparity was seen nationally in the evaluation of health insurance status. Only 53.7% of uninsured patients had an annual foot exam compared to 70% of insured patients. Examination of racial disparities for this metric across the nation revealed that patients identifying as “Black or African American only” and “American Indian or Alaska Native only” had the two highest percentages of people meeting the target at 75.8% and 72.8%, respectively [4].

This review highlights race, education, status of insurance, and location as important determinants of disparity in diabetes care. Provider-monitoring of diabetes progression, with regards to annual foot examinations and biannual HA1c testing in Arizona was disproportionate among different races. Black people, who make up 5.2% of Arizona’s population [6], did not receive standard diabetes care with regards to the above-mentioned metrics. On the other hand, American Indian and Alaska Native people, who make up 5.3% of Arizona’s population, surpassed the national target for annual foot examinations (76.7%) and nearly reached the national goal for biannual HA1c measurements (72.9%). Despite this apparent success, American Indian and Alaska Native people in Arizona experience the highest rate in diabetes-related death when compared to other races [2].

American Indian and Alaska Native people in Arizona have an even higher death-rate when compared to the national average [2]. One possible explanation for this disparity may be inadequate funding and intervention efforts. The United States spends half per capita for Native American and Alaska Native healthcare compared to spending for federal employees [7]. Access to care, cultural differences, poverty, and healthcare discrimination have been suggested as additional reasons for the lower life expectancy in this and other minority populations [8]. The National Institutes of Health implemented the Interventions for Health Promotion and Disease Prevention in Native American Populations in 2011 and have since reissued this initiative in an effort to address some of these barriers [7]. This provides some hope that diabetes-related death rates will fall and begin to reflect the improvements that have been made in diabetes management.

The second highest rate in diabetes-related death in Arizona are among individuals identifying as “Black or African American [2].” The increased disparity among patients who are Black, uninsured or have less than a high school education raise significant concern. As demonstrated by a growing body of literature, racial and ethnic minorities frequently suffer from health inequity. The disparate quality of care provided to two different minority groups in Arizona is noteworthy and deserves further examination. Attention from a public, as well as population, health perspective may reveal important differences in social determinants of health affecting these groups. It is also necessary to consider other contributing factors, including discrimination and implicit bias.

Identifying vulnerable populations, seeking to understand the root cause of why these groups experience health disparity, and analyzing results of prior public health initiatives are all critical to achieving health equity. Although public policy plays a crucial role in propelling change, the impact of the healthcare provider should not be underestimated. Patients with diabetes who report perceived discrimination have worse glycemic control and are less likely to receive HA1c testing [9]. Health professionals are encouraged to make health equity a priority and to consider the external forces affecting their patients’ wellbeing. Additionally, efforts to increase self-reflection and awareness of unconscious biases should be made. Implementing strategies to provide equitable care and to overcome social determinants of health at the level of the clinic, state and nation, can lead to improved health outcomes.

One limitation of this review is that data from HP 2020 were collected via survey and not medical health records. Of those surveyed, there was no further information in the data to show if patients had a primary care physician. Now that electronic health records are common, and data can be extrapolated more easily, it would be interesting to compare reported results between patients via survey and medical providers via health records. Patient survey results are dependent on health literacy and access to a telephone, and may be vulnerable to subjectivity. Additionally, each state conducts the BRFSS independently and thus methodologies may vary. In 2017, Arizona had over 15,000 responses [10] and the demographic breakdown of this population was similar to that of the state’s population. Information collected to assess both of the DM objectives reviewed in this article, were from the same group. As a result, perspectives of those surveyed should be consistent between questions. 

## Conclusions

Race and social determinants of health including, but not limited to, education and status of medical insurance are important factors that contribute to health. Uncontrolled diabetes is associated with poor quality of life and high cost. Identifying and addressing health disparities are necessary steps to reducing the burden of disease that DM imposes. Although DM affects people of all races, the results of this review indicate that patients with diabetes who are Black and live in Arizona received less provider-monitoring of their diabetes compared to other racial groups. Additionally, American Indian and Black populations experienced disproportionately high rates of diabetes-related death in Arizona. Accessing and analyzing public health data is critical to recognizing such inequities. Ongoing public health, as well as provider-based, strategies to address racial discrimination and social determinants of health are necessary. As HP 2020 comes to a close, and objectives are revised and added to HP 2030, attention to underrepresented minority groups should not be overlooked.
